# Clinical antidiabetic medication used in Alzheimer’s disease: From basic discovery to therapeutics development

**DOI:** 10.3389/fnagi.2023.1122300

**Published:** 2023-02-10

**Authors:** Juan Huang, Nanqu Huang, Di Cui, Jingshan Shi, Yu Qiu

**Affiliations:** ^1^Department of Pharmacology and Chemical Biology, Shanghai Jiao Tong University School of Medicine, Shanghai, China; ^2^Key Laboratory of Basic Pharmacology of Ministry of Education and Joint International Research Laboratory of Ethnomedicine of Ministry of Education, Zunyi Medical University, Zunyi, Guizhou, China; ^3^School of Public Health, Zunyi Medical University, Zunyi, Guizhou, China; ^4^The Third Affiliated Hospital of Zunyi Medical University (The First People’s Hospital of Zunyi), Zunyi, Guizhou, China

**Keywords:** Alzheimer’s disease, type 3 diabetes mellitus (T3DM), antidiabetic medication, type 2 diabetes mellitus, clinical research

## Abstract

Alzheimer’s disease (AD) is a common neurodegenerative disease. Type 2 diabetes mellitus (T2DM) appears to increase and contributing to the risk of AD. Therefore, there is increasing concern about clinical antidiabetic medication used in AD. Most of them show some potential in basic research, but not in clinical research. So we reviewed the opportunities and challenges faced by some antidiabetic medication used in AD from basic to clinical research. Based on existing research progress, this is still the hope of some patients with special types of AD caused by rising blood glucose or/and insulin resistance.

## Introduction

At present, Alzheimer’s disease (AD) lacks effective treatment methods and drugs. It is only delayed by some drugs that act on neurotransmitters (Marucci et al., 2021). In recent years, some progress has been made in anti-AD drugs, such as the Aducanumab approved by United States Federal Drug Administration (FDA), GV-971 approved by the National Medical Products Administration (NMPA), and Lecanemab, an initial decision on the drug’s approval by the FDA is expected by 2023, but they are all controversial (Karlawish and Grill, 2021; Xiao et al., 2021; The Lancet, 2022). The failure of a large number of drug studies on AD is largely related to the unknown pathogenesis of AD. Therefore, similar to the research of anti-tumor drugs, it is very promising to conduct more accurate subtype classifications for AD patients, and then conduct treatment drug research.

Epidemiological investigations of Type 2 diabetes mellitus (T2DM) and AD indicated that T2DM appears to increase and contributing to the risk of AD. Learning cognitive dysfunction, neuronal loss, etc., appear in T2DM patients (Noreen et al., 2018; Li et al., 2021). Further studies show they share lots of common link, including similar pathological features, etiology, targets, and involving same signaling pathways (Doherty et al., 2013; Chung et al., 2018; Zhang et al., 2018; Takeuchi et al., 2019; Gharibyan et al., 2020; Sun et al., 2020; Dekeryte et al., 2021; Liu et al., 2021; Yen et al., 2021). Thus, some researchers propose a theory that AD is Type 3 diabetes mellitus (T3DM) (Steen et al., 2005). Then, a growing number of studies have been carried out and showed that drugs for the treatment of T2DM also have certain improvement effect on AD (Akimoto et al., 2020). Most of them show some potential in basic research, but not in clinical research (Table 1). So we reviewed the opportunities and challenges faced by some antidiabetic medication used in AD from basic to clinical research. Based on existing research progress, this is still the hope of some patients with special types of AD caused by rising blood glucose or/and insulin resistance.

**TABLE 1:** Clinical antidiabetic medication used in AD.

Medication	Pre/clinical	Main results	References
MET	Preclinical	Improves spatial memory in diabetic mice, promotes the phagocytosis of pathological Aβ and Tau proteins by enhancing microglial autophagy capability, impaired glucose metabolism and mitochondrial dysfunction	Chiang et al., 2016; Chen et al., 2021; Oliveira et al., 2021
	Clinical	Reduces AD risk in the general population, beneficial effects on cognitive performance, long-term and high-dose metformin use is associated with a lower risk of incident AD in older people with diabetes	Sluggett et al., 2020; Pomilio et al., 2022; Zheng et al., 2022
	Clinical	The available evidence does not support the idea that MET reduces risk of AD, and it may increase the risk in Asians	Ha et al., 2021; Luo et al., 2022
GLP-1RA	Preclinical	Reduce the overproduction of Aβ and increase its removal, reduce hyperphosphorylation of Tau, neurofilaments, ameliorate mitochondrial dysfunction and prevents neuronal loss	Qi et al., 2016; Chen et al., 2017; Cai et al., 2018; An et al., 2019; Wiciński et al., 2019; Zhang et al., 2019; Zhou et al., 2019; Duarte et al., 2020; Jantrapirom et al., 2020; Xie et al., 2021
	Clinical	Administration of GLP-1 agonists may reduce the risk of AD in patients with T2DM	Akimoto et al., 2020
PPAR-γ agonists	Preclinical	Ameliorates Aβ deposition, controlling Aβ-induced dysfunctions of neuronal activity in the DG underlying memory loss in early AD	Jahrling et al., 2014; Toba et al., 2016; Badhwar et al., 2017; Hsu et al., 2017; Yang et al., 2017
	Clinical	PGZ (15–30 mg) has been demonstrated the greatest efficacy compared to placebo, subjects receiving RSG exhibits better delayed recall	Watson et al., 2005; Cao et al., 2018
	Clinical	Daily 0.8 mg oral PGZ does not significantly delay the onset of mild cognitive impairment due to AD, no evidence of clinically significant efficacy in cognition was detected for 2 or 8 mg RSG extended-release as adjunctive therapy	Harrington et al., 2011; Burns et al., 2021
DPP4i	Preclinical	DPP-4i drugs mainly improve inflammation and oxidative stress through the GLP-1/GLP-1R signaling pathway, affecting the production and clearance of toxic proteins	Kosaraju et al., 2013a,^2017^; Chen et al., 2019; Siddiqui et al., 2021
	Clinical	DPP-4i is associated with low amyloid burden and favorable long-term cognitive outcome in diabetic patients with ADCI, sitagliptin’s improvement of AD patient MMSE scores, vildagliptin to treatment improves the cognitive function of the older patients with T2DM	Isik et al., 2017; Ates Bulut et al., 2020; Jeong et al., 2021
SGLT2i	Preclinical	EMP ameliorate the cognitive deficits in APP/PS1xdb/db mice, GBC improves memory impairment with increasing insulin and reducing glucose and hippocampal inflammation in rats with T2DM	Lin et al., 2014; Esmaeili et al., 2020; Hierro-Bujalance et al., 2020
	Clinical	Long-term use of SGLT2i can improve cognitive function, especially for elderly diabetics	Wium-Andersen et al., 2019; Mui et al., 2021; Low et al., 2022; Mone et al., 2022

## Metformin

Metformin (MET) is a common oral anti-diabetic drug, which can lower blood glucose in many ways. Among these mechanisms regulated by MET to lower blood glucose, the regulatory mechanism centered on AMP-activated protein kinase (AMPK) plays an important role not only in diabetes mellitus (DM) but also in AD (Markowicz-Piasecka et al., 2017; Ma et al., 2022). In streptozotocin (STZ)-induced Swiss Webster mice, MET improves spatial memory in diabetic mice, which can be associated with reducing p-Tau and β-amyloid (Aβ) plaque load and inhibition of neuronal death (Oliveira et al., 2021). And in APP/PS1 mice, MET increases the level of p-AMPK and insulin degrading enzyme (IDE) protein in mice, and significantly reduces the Aβ level in the brain. Although it does not affect the enzyme activity of Aβ-related secretion enzymes (Lu et al., 2020). Additionally, in the APP/PS1 mouse injected Tau, MET can promote the phagocytosis of pathological Aβ and Tau proteins by enhancing microglial autophagy capability (Chen et al., 2021). But this performance shows a certain gender difference. In AβPP mice aged 12–14 months, MET activates AMPK to show a protective effect in female mice, but it shows a damage effect in male mice (DiTacchio et al., 2015). The results of these pre-clinical studies show that MET has a certain potential treatment effect on AD.

Although in many preclinical studies, MET shows an exciting role, the results in clinical studies are indeed unsatisfactory. First of all, there are still certain controversy in whether MET reduces the risk of AD. Studies have shown that MET can be a reduced AD risk in the general population (Zheng et al., 2022). But analyzing among Asians, MET has the risk of increasing the prevalence of AD (Ha et al., 2021; Luo et al., 2022). We believe that this is not consistent with the selection and analysis goals of data. To a large extent, the risk of AD population based on DM-based diseases can be considered as useful. We speculate that this is related to some AD patients without blood glucose changes. The AMPK activator represented by MET may be defined as a significant role in AD patients with T3DM. And some clinical studies have also verified our speculation. The use of MET does not increase the risk of AD. And long-term and large doses of MET are related to the risk of lowering AD with elderly DM (Sluggett et al., 2020). Therefore, we believe that we should have targeted design clinical trials to screen patients with abnormal blood glucose or have DM themselves, or early intervention for patients with DM merged mild cognitive impairment. And when considering the selection of drugs, the stage, type, and gender of the disease itself should be comprehensively considered.

## GLP-1 agonists

Glucagon-like peptide-1 (GLP-1) is one of the important targets for the treatment of diabetes. As an intestinal peptide, GLP-1 has glucose concentration dependent hypoglycemic effect *via* the potentiation of glucose-induced insulin secretion and the suppression of glucagon secretion (Keshava et al., 2017; Deacon, 2020). Moreover, numerous studies have demonstrated GLP-1 has potential neuroprotective and neurotrophic effects (Liu et al., 2021), so that GLP-1 based therapies may have favorable effects on AD. Such as liraglutide (LRGT), dulaglutide, lixisenatide, exenatide, and NLY01 have a significantly association with lowering risk of AD (Akimoto et al., 2020). The anti-AD effect of GLP-1 receptor (GLP-1R) agonist (GLP-1RA) has attracted the attention of researcher.

Liraglutide improves memory impairment in various AD models, decreasing AD-related insulin receptor (INSR), synaptic and Tau pathology in specific brain regions (Batista et al., 2018; Duarte et al., 2020). These effects involve multiple pathways. In Aβ, specifically, LRGT attenuates brain estradiol and GLP-1 and activates protein kinase A (PKA) levels, oxidative/nitrosative stress and inflammation in 11-month-old AD female mice, reduces their cortical Aβ_1–42_ levels (Duarte et al., 2020). LRGT can both reduce the overproduction of Aβ and increase its removal. One side, amyloid precursor protein (APP) is metabolized to Aβ by β-secretases and γ-secretases. LRGT decreases the formation of Aβ *via* inhibiting the activity of β-secretases and γ-secretases (Qi et al., 2016; Zhang et al., 2019; Jantrapirom et al., 2020). The other, binding to GLP-1R, LRGT activates the phosphoinositide-3 kinase/mitogen-activated protein kinase (PI3K/MAPK) dependent pathways, consequently following trafficking and clearing Aβ by increasing the presence of Aβ transporters in cerebrospinal fluid (Wiciński et al., 2019). In Tau, LRGT also reduces p-Tau, Aβ, *via* the protein kinase B/glycogen synthase kinase-3β (Akt/GSK-3β) pathways, reversing the p-INSR whose major downstream signaling molecules include insulin substrate 1, Akt and GSK-3β (Chen et al., 2017). At the same time, LRGT can reduce hyperphosphorylation of Tau, neurofilaments (NFs) and neuronal degeneration through restoring protein phosphatase-2A (PP2A) activity and altering in c-Jun N-terminal protein kainse (JNK) and extracellular regulated protein kinases (ERK) signaling apparently (Zhang et al., 2019; Jantrapirom et al., 2020). Additionally, LRGT ameliorates mitochondrial dysfunction and prevents neuronal loss with activation of the cAMP/PKA pathway in the brain of 5 × FAD mice. Activating the cAMP/PKA pathway, GLP-1 increases the p-DRP-1-s637 and mitigates mitochondrial fragmentation in Aβ-treated astrocytes. Then it further improves the Aβ-induced energy failure, mitochondrial reactive oxygen species (ROS) overproduction, mitochondrial membrane potential (MMP) collapse, and cell toxicity in astrocytes (Xie et al., 2021). In addition, Dulaglutide decreases the hyperphosphorylation of Tau and NFs proteins through improving the PI3K/Akt/GSK-3β signaling pathway (Zhou et al., 2019). Lixisenatide also plays an important role in memory formation, synaptic plasticity and cell proliferation of rats. It can reduce amyloid plaques, NFTs and neuroinflammation in the hippocampi of 12-month-old 3 × Tg female mice, which may be related to activating PKA-cAMP response element binding (CREB) signaling pathway and inhibiting p38-MAPK (Cai et al., 2018).

Generally speaking, these protection effects to a large extent rely on multiple pathways with regulatory regulation of insulin signal pathways as the core, thereby removing neurotoxic substances (Aβ and/or Tau). At the same time, it is difficult to define whether the control of inflammation and the protection of mitochondria is the cause or result. In addition, it is worth noting that a large number of preclinical studies on it come from China. And limited clinical research, it is difficult to prove the complex connection between correlation and clinical effectiveness. It is necessary to design more randomized controlled trial such as ELAD Study (Femminella et al., 2019). The clinical trials of it are worthy of attention. We look forward to these random dual-blind experiments that can have good results.

## PPAR-γ agonists

The peroxisome proliferator-activated receptor γ (PPAR-γ) is a prototypical ligand-activated nuclear receptor that coordinates lipid, glucose and energy metabolism. The PPAR-γ agonists have emerged as potent insulin sensitizers used in the treatment of T2DM. Pioglitazone (PGZ) is a member of the thiazolidinedione (TZD) family. In a pre-clinical study, it improves cognitive deficits in AD animal models by reducing Aβ levels. And it normalizes the p35 protein and p-CRMP2 levels in the cerebellum, ameliorates impaired motor coordination ability and long-term depression (LTD) in APP/PS1 mice at the pre-Aβ accumulation stage (Toba et al., 2016). It also enhances peripheral and brain insulin sensitivity in diet-induced insulin resistance model rats, ameliorates Aβ_1–42_ deposition in the hippocampus by increasing IDE and PPARγ expression. Notably, activating the PI3K/Akt/GSK-3β pathway is also demonstrated to serve a role in PGZ-induced Aβ_1–42_ degradation, which is abrogated by the PPARγ antagonist GW9662 (Yang et al., 2017). Furthermore, PGZ treatment could inhibit Cdk5 activity by decreasing p35 protein level. More importantly, PGZ corrects long-term potentiation (LTP) deficit caused by Aβ exposure in cultured slices and rescues impaired LTP and spatial memory (Badhwar et al., 2017). Although clinical studies have shown that PGZ has the potential of AD for treatment, the results of clinical trials are indeed unsatisfactory. Daily 0.8 mg oral PGZ did not significantly delay the onset of mild cognitive impairment due to AD (Burns et al., 2021). Interestingly, PGZ 15–30 mg demonstrates the greatest efficacy compared to placebo in network meta-analysis (Cao et al., 2018).

The rosiglitazone (RSG) improves hippocampus-dependent cognitive deficits in some AD patients and ameliorates deficits in the Tg2576 mouse for AD amyloidosis (Jahrling et al., 2014). Then the research further verified RSG treatment rescues cognitive deficits and reduces aberrant activity of granule neurons in the dentate gyrus (DG) (Hsu et al., 2017). Clinical trials of RSG have shown some contradictions. Early studies showed some anti-AD potential of RSG (Watson et al., 2005), while subsequent clinical trials fail to achieve the desired results (Harrington et al., 2011). Therefore, in clinical trials on RSG, screening for multiple subgroups in the AD patient population and enrolling patients using predictive biomarkers has received attention (O’Bryant et al., 2021). We speculate that with the further development of AD typing and biomarkers, such studies may bring new hope.

## DPP-4 inhibitors

Different from GLP-1 agonists, dipeptidyl peptidase 4 inhibitors (DPP4i) do not possess inherent glucose-lowering activity. It inhibits the activity of the enzyme DPP4, then it decreases blood glucose level through GLP1 to treat T2DM (Stoian et al., 2020). DPP-4i contains saxagliptin, vildagliptin, linagliptin, sitagliptin. They have beneficial effects on amyloid aggregation and longitudinal cognitive outcome in diabetic AD-related cognitive impairment (ADCI) (Jeong et al., 2021). However, the mechanism by which they work seems different.

Sitagliptin has been demonstrated to have antioxidative and antiapoptotic properties by modifying glutamate and glutathione levels within the region of hippocampus in mice (El-Sahar et al., 2015). Meanwhile, it increases the synaptic proteins and the O-Glycosylation (Chen et al., 2019). Moreover, sitagliptin improves the impaired cognitive by the potential mechanisms that regulating neuroinflammation, antioxidation, and antiapoptotic, and the level of GLP-1 and GLP-1R (Wiciński et al., 2018). Finally achieve the goal of protecting learning and memory. Interestingly, preliminary clinical results show that sitagliptin’s improvement of AD patient mini-mental state examination (MMSE) scores is better than MET (Isik et al., 2017). With a higher selectivity, saxagliptin has the same effect as sitagliptin that protect learning and memory through GLP-1/GLP-1R signaling pathway (Kosaraju et al., 2013a; Chen et al., 2019). Like sitagliptin, linagliptin treatment mitigates the cognitive deficits that attributed to the improvement of incretin levels and attenuate Aβ, p-Tau and neuroinflammation in the brain mice of 3 × Tg-AD and Aβ_1–42_ induced rat model of AD (Kosaraju et al., 2017; Siddiqui et al., 2021). Moreover, linagliptin can ameliorate cognitive deficits through insulin pathway (Siddiqui et al., 2021) and restore the impaired insulin signaling caused by Aβ in neuronal cells (Kornelius et al., 2015). Vildagliptin also demonstrates a unique mechanism for Aβ and Tau clearance and reverses the cognitive deficits and pathology observed in AD possibly *via* modulating Klotho protein together with Akt pathway (Kosaraju et al., 2013b; Yossef et al., 2020). The addition of vildagliptin to treatment improved the copying subdomain of cognitive function and metabolic control of the older patients with T2DM (Ates Bulut et al., 2020).

These results indicate that DPP-4i drugs mainly improve inflammation and oxidative stress through the GLP-1/GLP-1R signaling pathway, affecting the production and clearance of toxic proteins, thereby improving cognitive function. But most of the studies are basic research, although there are a small number of clinical studies on sitagliptin and vildagliptin in cognition, but they are all preliminary and short-term, and the sample size is small. Our suggestion would be best to carry out the anti-AD research of DPP-4i after a breakthrough in the anti-AD research of GLP-1/GLP-1R or the combination of DP-4i and the first approved effective anti-AD drug.

## SGLT2 inhibitors

Sodium glucose cotransporter 2 inhibitors (SGLT2i) can reduce blood glucose by inhibiting its reabsorption in proximal tubules and by promoting urinary glucose excretion. A growing numbers evidence indicates that SGLT2i such as empagliflozin (EMP), canagliflozin, dapagliflozin, ertugliflozin, and sotagliflozin have neuroprotective potential in a murine mixed model of T2DM and AD (Lin et al., 2014; Rizvi et al., 2014; Shaikh et al., 2016; Hierro-Bujalance et al., 2020).

Empagliflozin help to limit cortical thinning and reduce neuronal loss, hemorrhage, microglia burdens and SPs burden, also improves cerebral microvascular eventually ameliorate the cognitive deficits in APP/PS1xdb/db mice (Lin et al., 2014; Hierro-Bujalance et al., 2020). Dapagliflozin and invokana might act as potent dual inhibitors of SGLT2 and AchE, which contributes to cognitive improvement, as well as ertugliflozin and sotagliflozin (Rizvi et al., 2014; Shaikh et al., 2016). Glibenclamide (GBC) treatment improves memory impairment with increasing insulin and reducing glucose and hippocampal inflammation in rats with T2DM and sporadic AD (Esmaeili et al., 2020). And SGLT2i exert anti-inflammatory and antioxidant effects at the cellular level mainly *via* regulation of the molecular target of rapamycin (mTOR) pathway, which could ameliorate the progression of AD (Esterline et al., 2020; Katsenos et al., 2022). And in nested case control study evaluating diagnoses of dementia in patients with T2DM, SGLT2i use showed a 42% reduction in dementia risk (Wium-Andersen et al., 2019). Interestingly, in the population-based cohort study of T2DM patients treated with SGLT2i and DPP4i, the use of SGLT2i is associated with lower risks of dementia, compared with DPP4i (Mui et al., 2021). And a prospective study shows significant beneficial effects of the EMP on cognitive in frail older adults with diabetes (Mone et al., 2022). In addition, SGLT2I’s ≥ 3 years use is related to the improvement of cognitive scores (Low et al., 2022). According to the existing evidence, long-term use of SGLT2i can improve cognitive function, especially for elderly diabetics. However, the role of AD patients still needs further study.

## Conclusion

Based on the facts that T2DM and AD share common features, drugs used to treat T2DM are being investigated for efficacy in AD. Consequently, studies on drugs used for T2DM in AD found these treatments may represent a promising approach to fight AD, which include MET, GLP-1RA, PPAR-γ agonists, DPP-4i and SGLT2i (Cao et al., 2018). However, there are differences in their effects in basic and clinical research on anti-AD (Table 1). At the same time, the anti-AD effect of insulin is also controversial, but there are too many studies involved, so this review will not discuss it for the time being. We believe that the difference between the results of clinical antidiabetic medication in anti-AD treatment clinical trials and basic experiments is mainly related to the following: (1) We speculate that they are not effective for all types of AD, but may be a special type: AD patients who also suffer from diabetes. They may even be useful only for cognitive dysfunction caused by insulin resistance. (2) These effects interact with the improvement of insulin resistance, so perhaps early intervention may have a better effect. (3) Complex and interactive-oxidation, anti-neuroinflammation, and improve energy metabolism play an important role in it, so the combination of drugs to treat AD may have more potential. Such drugs are not a very good solution under the existing evidence conditions. Looking forward to more refined pathological research on AD classification, it may rekindle hope for the clinical research of such drugs.

But screening subjects based on more subtypes, or recruiting patients using predictive biomarkers, would severely narrow the pool of subjects who ultimately meet inclusion criteria and would substantially increase the cost of clinical trials. Unless a reasonable combination of predictive biomarkers can be found, or there is a well-defined classification of AD subtypes. Otherwise, it will still be a bottomless pit to rush to carry out relevant and more refined clinical trials, and it is not worth investing too much energy. Moreover, hypoglycemia, the side effect of such drugs, is still not negligible. In the elderly, falls caused by hypoglycemia often cause serious consequences. Therefore, we have to consider the scope of application of this type of drug and the direction that needs to be considered in the design of such drugs. It is best to regulate the insulin pathway and have little effect on blood glucose (or be able to control blood glucose stably within a reasonable range). Weighing the pros and cons is an unavoidable multiple-choice question in drug development. In addition to genes, diabetes is often closely related to eating habits, and intestinal flora also play a key role in it. Whether these drugs affect the intestinal flora and thus affect AD is also an aspect worthy of attention. It is also worth noting that in the absence of strong evidence-based medical evidence, the use of hypoglycemic drugs for the prevention and treatment of AD will face many risks.

In the current situation, we should not be pessimistic. While looking forward to the progress of basic research on AD, we should more actively conduct group statistics or subtype analysis on existing failed clinical trials, especially large-sample clinical trials. Not only may there be unexpected surprises, but it will also play a guiding role in the development of future clinical trials.

## Author contributions

All authors listed have made a substantial, direct, and intellectual contribution to the work, and approved it for publication.
